# Screening for and Managing the Person with Frailty in Primary Care: ICFSR Consensus Guidelines

**DOI:** 10.1007/s12603-020-1498-x

**Published:** 2020-10-17

**Authors:** J. G. Ruiz, E. Dent, John E. Morley, R. A. Merchant, J. Beilby, J. Beard, C. Tripathy, M. Sorin, S. Andrieu, I. Aprahamian, H. Arai, M. Aubertin-Leheudre, J. M. Bauer, M. Cesari, L.-K. Chen, A. J. Cruz-Jentoft, P. De Souto Barreto, B. Dong, L. Ferrucci, R. Fielding, L. Flicker, J. Lundy, J. Y. Reginster, L. Rodriguez-Mañas, Y. Rolland, A. M. Sanford, A. J. Sinclair, J. Viña, D. L. Waters, C. Won Won, J. Woo, B. Vellas

**Affiliations:** 1 Geriatric Medicine, University of Miami Miller School of Medicine, Miami, Florida USA; 2 Adelaide and Baker Heart and Diabetes Institute, Torrens University of Australia, Melbourne, Australia; 3 Division of Geriatric Medicine, Saint Louis University School of Medicine, St. Louis, Missouri USA; 4 Division Geriatric Medicine, Department of Medicine, National University Hospital, National University Health System, Singapore, Singapore; 5 Think Education, Torrens University, Adelaide, Australia; 6 Centre of Excellence in Population Ageing Research, University of New South Wales, Sydney, Australia; 7 Primary Care Center and Division of General Medicine, University of Texas Health San Antonio, San Antonio, Texas USA; 8 University of Florida College of Medicine, Gainesville, Florida USA; 9 Department of Epidemiology, UMR1027 Inserm — Toulouse University III, University Toulouse, Toulouse, France; 10 Department of Internal Medicine, Division of Geriatric Medicine, Jundiaí Medical School, Sao Paulo, Brazil; 11 University Medical Center Groningen, Department of Psychiatry, University of Groningen, Groningen, The Netherlands; 12 National Center for Geriatrics and Gerontology, Obu, Japan; 13 Dept des Sciences de l’activité physique, CRIUGM, Université du Québec à Montréal, Montréal, QC Canada; 14 Center for Geriatric Medicine and Network Aging Research, Heidelberg University, Agaplesion Bethanien Krankenhaus, Heidelberg, Germany; 15 IRCCS Istituti Clinici Scientifici Maugeri, University of Milan, Milano, Italy; 16 Center for Geriatrics and Gerontology, Taipei Veterans General Hospital, Taipei, Taiwan; 17 Aging and Health Research Center, National Yang Ming University, Taipei, Taiwan; 18 Servicio de Geriatria, Hospital Universitario (IRYCIS), Ramón y Cajal, Madrid, Spain; 19 Gérontopôle of Toulouse, Institute of Ageing, Toulouse University Hospital (CHU Toulouse) and UPS/Inserm UMR 1027, University of Toulouse, Toulouse, Toulouse France; 20 National Clinical Research Center for Geriatrics, West China Hospitals of Sichuan University, Chengdu, China; 21 National Institute on Aging/NIH, Baltimore, Maryland 21224 USA; 22 Exercise Physiology, and Claude C. Pepper Older Americans Independence Center, Jean Mayer USDA, Human Nutrition Research Center on Aging at Tufts University, Boston, Massachusetts USA; 23 Western Australia Centre for Health and Ageing Medical School, University of Western Australia, Perth, Australia; 24 Perry County Memorial Hospital, Perryville, Missouri USA; 25 Department of Public Health, Epidemiology and Health Economics, and Chair for Biomarkers of Chronic Diseases, Biochemistry Department of College of Sciences, King Saud University, Riyadh, Saudi Arabia; 26 University of Liege, Liege, Belgium; 27 Hospital Universitario de Getafe, Servicio de Geriatria, Madrid, Spain; 28 Service de Médecine Interne et Gérontologie, Gerontopole, Toulouse France; 29 Foundation for Diabetes Research in Older People, King’s College, London, UK; 30 Dept Physiology Faculty Medicine, University of Valencia, Valencia, Spain; 31 Department of Medicine and School of Physiology, University of Otago, Dunedin, New Zealand; 32 Elderly Frailty Research Center, Department of Family Medicine, College of Medicine, Kyung Hee University, Seoul, South Korea; 33 Department of Medicine, the Chinese University of Hong Kong, Hong Kong, China; 34 Division of Geriatric Medicine, Saint Louis University, SLUCare Academic Pavilion, Section 2500, 1008 S. Spring Ave., 2nd Floor, St. Louis, MO 63110 USA

## Introduction

Frailty is now a well-recognized and common syndrome among older persons (1–3). Frailty is a syndrome which increases the risk of an older person to develop disability or to die when exposed either to physical or psychosocial stressors (4, 5). Although frailty, disability and multimorbidity often coexist and interact, they are distinct and separate concepts (6). Growing evidence suggests that each of these interrelated conditions is preventable and their associated complications manageable (6–8). However, early identification is imperative as once disability and multimorbidity occur, frailty in less likely to be prevented or reversed (9–11). As such it should be distinguished from persons with disability in their activities of daily living. The conditions leading to the frailty syndrome should have some degree of reversibility, thus distinguishing it from multimorbidity (7, 8, 12). Recently, the International Conference of Frailty and Sarcopenia Research (ICFSR) formulated evidence-based guidelines for the identification and management of physical frailty (13). Physical frailty was originally defined and validated by Fried et al (12, 14). This definition included measurements of low activity level, slowness of walking, muscle weakness, exhaustion and weight loss. This approach differs from that of Rockwood and Mitnitski (15) which used the number of “deficits” (signs, symptoms, clinical conditions) to determine a frailty index. Primary care represents the entry point into the health care system for many older adults who may be pre-frail and frail. A shortage of geriatricians and the higher frequency of frailty in community settings call for primary care clinicians (general practitioners, generalists, family physicians) to increasingly assess and manage older adults at risk for frailty or who are already frail.

The purpose of this paper is to suggest practical frailty screening and management strategies in primary care settings. We will also discuss the characteristics of these instruments and their applicability to primary care. For the sake of consistency hereafter, we will refer to clinicians delivering primary care as primary care providers.

## Screening (Case Finding)

Primary care providers around the world report high patient workloads. The average primary care physician spends between less than a minute on consultations in Bangladesh to over 20 minutes in Sweden (16). Less than half of these physicians spend more than 10 minutes for consultations. The short amount of time physicians spent with older persons makes it extremely difficult to identify and develop a comprehensive diagnostic and management plan for geriatric syndromes. Primary care providers need easy and rapid approaches to help them identify patients with frailty. Below we describe time-efficient and validated screening tools that clinicians can use to identify frailty in older persons in primary care.

**Figure 1:**
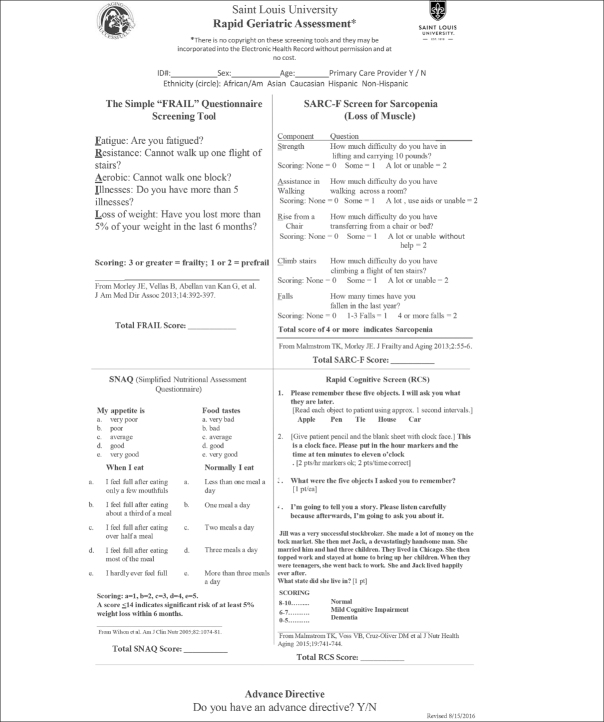
The FRAIL and Other Components of the Rapid Geriatric Assessment

The FRAIL scale (Figure 1) is a simple 5-item questionnaire that can be answered in 15 to 30 seconds (17, 18). In persons over 50 years of age the FRAIL Scale predicted disability and mortality at 9 years (19, 20). It performed as well as the Fried Frailty Phenotype (16a) and the Study of Osteoporosis Fractures (SOF). In the Australian Longitudinal Study on Women’s Health, the FRAIL scale predicted future disability over a 15-year period in middle aged women (21). A large study in Hong Kong demonstrated that FRAIL predicted over 4 years both disability and mortality as well as the CHS scale and the Rockwood Frailty Index (22). FRAIL predicted mortality in the Survey of Health Aging and Retirement in Europe (SHARE) (23). Numerous other studies have validated the predictive capacity of FRAIL (24–28). Thus, the FRAIL scale is now recommended as a screen tool for older persons visiting primary care providers in Australia (29) and in Brazil (30). An adapted version of the tool has also been developed for nursing homes (i.e., FRAIL-NH), which has shown to be predictive of adverse outcomes in the long term care setting (31, 32).

Another rapid screening test for frailty is the Clinical Frailty Scale (CFS) (33–35). The CFS scale consists of 9 items and is available in a pictorial version with corresponding text. It is correlated with the Frailty Index and is predictive of mortality (33, 36). The first three items refer to persons that are non-frail, item four assesses vulnerability whereas items five to eight include an assessment of disability. It is uncertain how correctly the average clinician can classify persons in the different categories (especially distinguishing frail from the disabled) by using the Clinical Frailty Scale (CFS) and without falling into the risk of subjectivity.

The Vulnerable Elders Survey-13 (VES-13) consists of questions to recognize older persons with frailty (37, 38). The VES-13 questionnaire consists of items measuring activities of daily living, physical function, self-rated health, and one question on age. It is a practical and brief screening tool that can be staff-administered or self-administered in less than 5 minutes. It has been demonstrated to be a good predictor of decreased function and death in older persons (39, 40).

The Kihon checklist was introduced by the Japanese long-term care insurance system in 2006 as an evaluation of frailty (41, 42). It consists of 25 yes or no questions that evaluate the domains of physical function, nutrition, feeding, social activity, memory, mood and lifestyle (43). It has been validated against the Fried frailty phenotype (41). The Kihon checklist is predictive of mortality (44) and shows good diagnostic accuracy in identifying frailty in primary care based on a recent Australian study (45).

The VES-13 (37, 38) and Kihon checklist (41, 42) include items assessing basic and instrumental activities of daily living among their scoring items. As with the Clinical Frailty Scale, clinicians using these instruments may have difficulties at distinguishing frailty from disability.

The World Health Organization (WHO) has focused on developing an approach to screen persons for decreases in intrinsic capacity, defined as “the combination of the individual’s physical and mental, including psychological, capacities” (46). To screen for loss of intrinsic capacity they have developed the “Integrated Care for Older People” (ICOPE) instrument (47, 48). Primary care providers should match for frailty development due to physical inactivity during the COVID-19 pandemic (47). While not specifically designed to identify frailty and having no designated cutoff to distinguish frailty states, the screening test can be delivered by a professional screener or by patient self-assessment using either a mobile application (App) or the BOTFRAIL (an internet conversational robot). The ICOPE screening test consists of 6 areas including measurements of cognition, mobility, malnutrition, vision impairment, hearing loss and depression. (Table 1)

**Table 1:** Screening Tool for the “Integrated Care for Older Persons” (ICOPE)

**Priority Conditions Associated with Declines in Intrinsic Capacity**	**Tests**
Cognitive Decline	1. Remember three words: Flower, door, rice (for example)
	2. Orientation in time and space: What is the full date today? Where are you now (home, clinic, etc.)?
	3. Recalls the three words?
Limited Mobility	Chair rise test: Rise from chair five times without using arms. Did the person complete five chair rises within 14 seconds?
Malnutrition	1. Weight loss: Have you unintentionally lost more than 3 kg over the last three months?
	2. Appetite loss: Have you experienced loss of appetite?
Visual Impairment	Do you have any problems with your eyes: Difficulties in seeing far, reading, eye diseases or currently under medical treatment (e.g., diabetes, high blood pressure)?
Hearing Loss	Hears whispers (whisper test) or Screening audiometry result is 35 dB or less or Passes automated app-based digits-in-noise test
Depressive Symptoms	Over the past two weeks, have you been bothered by
	• Feeling down, depressed or hopeless?
	• Little interest or pleasure in doing things?

The Study of Osteoporotic Fractures (SOF) frailty scale was developed and validated in an all-female cohort. It consists of three items that are easy to administer: the ability to rise from an armless chair five times (inability = 1); response to the question “Do you feel full of energy?” (answer of “no” = 1); and weight loss > 5% in the past year (presence of weight loss = 1). Each item is scored as 0 for normal or 1 for abnormal (Prefrail =1, and Frail = 2 or 3) (49)». The SOF can be easily incorporated into a primary care practice and is useful in the identification of patients who may require referral for comprehensive geriatric assessment.

Frailty indexes that are automatically generated from electronic health records or administrative claims data may offer distinct advantages to busy primary care providers. As electronic health records become increasingly ubiquitous in primary care practices in high income countries, clinicians can use this information at the point of care to identify patients with frailty. Recently developed electronic frailty indexes have demonstrated predictive validity for hospitalizations, nursing home placement, cost of care, prediction and resource allocation to care for populations in value-based care delivery (50–53). A limitation is that electronic health records may not yet be widely available in many low- and middle-income countries. Furthermore, they might rely on medical data of limited relevance for the older person, and ignore aspects of critical importance in geriatric patients (e.g., functional status).

## Referral to Comprehensive Geriatric Assessment (CGA)

Investigators have often validated frailty screening instruments against the CGA (54). Screening instruments serve to identify those older adults who may be at risk for frailty or may have already developed frailty. Although many frailty screening instruments are sensitive, these tools often display low specificity (55). Thus, screening tests require confirmation of frailty with more thorough evaluations of the older person such as those part of a CGA. Geriatric assessment may uncover previously unrecognized problems that may contribute to the development or progression of frailty in older adults (56–58). Timely identification of these problems may lead clinicians to design and implement personalized interventions which can improve patient outcomes (57, 58). At the same time, it is important to remind that the CGA is a process diagnostically and therapeutically. The assessments conducted in the first part of the CGA to identify the persons critical aspects should always be followed by a multi-disciplinary and integrated intervention to make the methodology meaningful.

## Management of Frailty

There is a growing evidence in support of a variety of interventions that target older adults with frailty in primary care settings. Research indicates that exercise, nutrition and geriatric assessment represent effective, evidence-based interventions in primary care. A recent meta-analysis of 31 studies including 4794 participants concluded that resistance exercise, with or without nutrition supplementation may improve the frailty status of older adults in primary care settings. In older subjects with diabetes and frailty, resistance exercise as part of a multimodal approach significantly improved physical performance over one year measured by the short performance physical battery (SPPB) which was accompanied by a significant decrease in healthcare expenditure (59). Comprehensive Geriatric Assessment was also more effective than control groups at reducing frailty (58). Older adults with frailty often display prolonged periods of sedentary behaviors (60). Interventions to reduce overall sedentary behavior in older people with frailty may include short bouts of physical activity after intervals of uninterrupted inactivity (13, 31). Although less studied, other clinical interventions such as nutrition may offer benefits to older adults with frailty in outpatient settings. Observational studies suggest potential benefits of the Mediterranean diet (61, 62) and of vitamin D supplementation in patients that are deficient (63, 64). A summary of these recommendations can be seen in Table 2.

**Table 2:** Management of Frailty in Primary Care

**Primary Prevention**	**Secondary Prevention**	**Tertiary Prevention**
1. Provide community education including television, newspapers, magazines and social media to do aerobic and resistance exercise regularly 2. Health care professionals to regularly reinforce the importance of exercise. 3. Community lectures by health care professionals on the importance of exercise 4. Yearly screening with a rapid screen for frailty (FRAIL or ICOPE)	If positive frailty screen: 1. Check for and treat possible reversible causes as in Table 1 or 2 2. Enroll in an exercise program 3. Advise on adequate (leucine enriched) protein intake 4. Consider grip strength, 4m gait speed and short physical performance battery	1. Check ADLs and IADLs 2. Refer for comprehensive geriatric assessment 3. Refer to physical and occupational therapy 4. Optimize home environment 5. Provide a long term exercise program

The following sections give an overview of two examples of management approaches implemented in primary care settings.

The Rapid Geriatric Assessment: A management program for the different components of the FRAIL has been developed at Saint Louis University and is being developed into an App (20, 22). For fatigue, common causes are depression, sleep apnea, hypotension, anemia, hypothyroidism, hypoxia and vitamin B12 deficiency (65). Persons who have trouble completing the resistance and aerobic questions can be referred to multicomponent exercise program for sarcopenia (49, 66). They may also benefit from a leucine enriched essential amino acid supplement (67). Persons with more than five illnesses should have their medications reviewed to see if they are on inappropriate medications for older persons or if they have polypharmacy, where reduction of some medicines may improve their function (68–71). Older persons with weight loss should be examined for treatable causes of weight loss as delineated by the MEALS-ON-WHEELS mnemonic (8). In addition, the use of a caloric supplement can be considered (72) (Table 3). The FRAIL screen has been integrated with 3 other tests: The SARC-F (Sarcopenia) (2, 73), the Simplified Nutrition Appetite Questionnaire (SNAQ) (74, 75) and the Rapid Cognitive Screen (RCS) (76) to provide a more comprehensive geriatric examination, which can be performed by a primary care provider or other allied health care professionals (Figure 1). The complete RGA can be carried out in under 5 minutes (77) and is available as an App which was utilized by the National University Health System in Singapore (78). Furthermore, the RGA can be integrated into the Medicare Annual Wellness Visit (79).

**Table 3:** Diagnostic and Management Program for an Older Individual who has Deficits on the FRAIL Questionnaire (Copyright Saint Louis University and John E. Morley)

**Potential Deficits**
Fatigue: Exclude Depression
Exclude Sleep Apnea
Measure TSH, Vitamin B^12^ and Hemoglobin
Exclude low blood pressure or orthostasis resistance or Aerobic: Aerobic and Resistance exercise
Leucine enriched essential amino acid supplement
Measure bioavailable vitamin D and replace if low
Illnesses: Remove inappropriate medications including those causing side effects
Reduce Polypharmacy
Loss of Weight: Exclude depression
Stop drugs causing weight loss
Check for elderly abuse
Is the person paranoic (late life paranoia) or afraid being overweight will kill them?
Does the person have dysphagia?
Are there oral problems making chewing difficult?
Does the person have a nosocomial infection, e.g., *Helicobacter pylori* or tuberculosis?
Does the person have dementia?
Does the person have hyperthyroidism, Addison’s disease or pheochromocytoma?
Does the person have celiac disease or pancreatic insufficiency?
Does the person have eating difficulties?
Is person on low salt, low cholesterol or other therapeutic diet?
Does the person have cholecystitis?

The Integrated Care for Older People: The ICOPE program may be indicated for older persons that are either pre-frail or frail. The ICOPE rationale to target older persons at the pre-frail stage is that early interventions aimed at reversing pre-frailty or preventing the patient from becoming frail are more likely to be successful. It may also reduce the need to implement a higher number of step by step approaches which may be more suited to older persons who are already frail. In the ICOPE program, the older person is referred to either a primary care provider or a trained nurse to complete a geriatric assessment that includes a personalized intervention plan which is reassessed every 4 months. The follow up reassessments can be performed remotely through telemedicine (80). Each time the team detects a worsening of one or more ICOPE functions, they proceed to evaluate the reasons for the deficit (step 2 ICOPE) and propose personalized interventions (step 3). The ICOPE program encompasses medical, environmental and social domains. Moreover, older persons participation and empowerment are integral parts of the ICOPE program. Older persons learn how to self-assess their ICOPE functions using self-managements tools, apps or conversational bots (automated computer programs that interact with humans) (80). Digital medicine, e-health and telemedicine technologies offer healthcare teams efficient ways to monitor ICOPE functions and intervene in a timely fashion when indicated. For example, as part of the INSPIRE program a nurse monitors older persons’ functional status by reviewing databases every 4 months. If new abnormalities are detected, the nurse refers the older person to the primary care provider for step 2. The primary care provider can then choose to implement step 2 part during a routine clinical encounter, ask a trained nurse to perform a more comprehensive geriatric assessment, or contact a geriatrician for a tele-expertise consultation. The primary care provider uses the results of the cognitive and frailty scales to decide whether it is appropriate to refer the patient to a geriatrician (48, 80, 81). Another possible approach is to utilize the Korean Frailty Index for primary Care (82).

## Frailty within a Primary Care Model of Care

The optimal management of an older person with frailty in primary care requires a coordinated and integrated approach. Primary care providers need to work in collaboration with multidisciplinary teams which involve geriatricians, allied health professionals (including physiotherapists, dieticians, exercise physiologists, social workers, and occupational therapists), caregivers and the patient themselves. A model of care that is widely adopted in the US is the patient-centered medical home model (PCMH) (83). The principles that guide this model are relevant to the care of older adults with frailty ensuring the delivery of comprehensive care, that is patient-centered, coordinated, accessible, safe and of high quality (84, 85). The PCMH model may provide an organizing framework for the implementation of screening and management strategies by primary care providers (86, 87). Within this model, primary care providers lead a team of professionals to ensure comprehensive and coordinated care for older adults with frailty.

## The Role of Education and Training

Key to the success of frailty screening and management initiatives in primary care is participation of competent and motivated primary care providers (88). Education and training of the workforce represent crucial approaches to increase the uptake of screening and management for frailty in primary care (89). Success of these initiatives will demand that undergraduate, graduate and continuing professional development training programs for medical and allied health practitioners include these topics in their curricula.

## Conclusion

A number of rapid screening tests have been developed to evaluate frailty in the older population. These tests are predictive of poor clinical outcomes. Screening and managing frailty appear to be reasonable approaches to reducing disability in older persons. It is important to adapt our health care system to the aging of the population and move from the traditional disease-oriented medical model to a more global and modern patient-centered model that encompasses the assessment, monitoring and maintenance of function with the ultimate goal of preventing frailty and disability.
